# Alternative splicing of the human gene SYBL1 modulates protein domain architecture of longin VAMP7/TI-VAMP, showing both non-SNARE and synaptobrevin-like isoforms

**DOI:** 10.1186/1471-2199-12-26

**Published:** 2011-05-24

**Authors:** Marcella Vacca, Lara Albania, Floriana Della Ragione, Andrea Carpi, Valeria Rossi, Maria Strazzullo, Nicola De Franceschi, Ornella Rossetto, Francesco Filippini, Maurizio D'Esposito

**Affiliations:** 1 Institute of Genetics and Biophysics "A.Buzzati Traverso" Consiglio Nazionale delle Ricerche, via P. Castellino 111, 80131 Naples, Italy; 2 Molecular Biology and Bioinformatics Team "MOLBINFO", Department of Biology, University of Padua, viale G. Colombo 3, 35131 Padova, Italy; 3 IRCSS, INM Neuromed, 86077 Pozzilli, Italy; 4 Department of Biomedical Sciences, University of Padua, viale G. Colombo 3, 35131 Padova, Italy; 5 Institute for Animal Production System in Mediterranean Environment Sciences, Consiglio Nazionale delle Ricerche, via Argine 1085, 80147 Naples, Italy; 6 Institute of Neurosciences, Consiglio Nazionale delle Ricerche, viale G. Colombo, 3, 35131 Padova, Italy

## Abstract

**Background:**

The control of intracellular vesicle trafficking is an ideal target to weigh the role of alternative splicing in shaping genomes to make cells. Alternative splicing has been reported for several Soluble *N*-ethylmaleimide-sensitive factor Attachment protein REceptors of the vesicle (v-SNAREs) or of the target membrane (t-SNARES), which are crucial to intracellular membrane fusion and protein and lipid traffic in Eukaryotes. However, splicing has not yet been investigated in Longins, i.e. the most widespread v-SNAREs. Longins are essential in Eukaryotes and prototyped by VAMP7, Sec22b and Ykt6, sharing a conserved N-terminal Longin domain which regulates membrane fusion and subcellular targeting. Human VAMP7/TI-VAMP, encoded by gene SYBL1, is involved in multiple cell pathways, including control of neurite outgrowth.

**Results:**

Alternative splicing of SYBL1 by exon skipping events results in the production of a number of VAMP7 isoforms. In-frame or frameshift coding sequence modifications modulate domain architecture of VAMP7 isoforms, which can lack whole domains or domain fragments and show variant or extra domains. Intriguingly, two main types of VAMP7 isoforms either share the inhibitory Longin domain and lack the fusion-promoting SNARE motif, or vice versa. Expression analysis in different tissues and cell lines, quantitative real time RT-PCR and confocal microscopy analysis of fluorescent protein-tagged isoforms demonstrate that VAMP7 variants have different tissue specificities and subcellular localizations. Moreover, design and use of isoform-specific antibodies provided preliminary evidence for the existence of splice variants at the protein level.

**Conclusions:**

Previous evidence on VAMP7 suggests inhibitory functions for the Longin domain and fusion/growth promoting activity for the Δ-longin molecule. Thus, non-SNARE isoforms with Longin domain and non-longin SNARE isoforms might have somehow opposite regulatory functions. When considering splice variants as "natural mutants", evidence on modulation of subcellular localization by variation in domain combination can shed further light on targeting determinants. Although further work will be needed to characterize identified variants, our data might open the route to unravel novel molecular partners and mechanisms, accounting for the multiplicity of functions carried out by the different members of the Longin proteins family.

## Background

The human gene SYBL1 (synaptobrevin-like 1) expression is finely regulated at different layers. Even if located in the Xq/Yq pseudoautosomal region, SYBL1 is subject to છ inactivation [1] and its allelic expression is controlled by multiple epigenetic mechanisms [2]. Moreover, SYBL1 expression is altered in human pathologies characterized by DNA methylation derangement [3], such as ICF syndrome [4,5] and hyperhomocysteinemia [6]. SYBL1 encodes the v-SNARE protein VAMP7, an important modulator of intracellular trafficking also known as Tetanus neurotoxin Insensitive VAMP (TI-VAMP) [7]. Soluble *N*-ethylmaleimide-sensitive factor attachment protein receptors (SNAREs) of the vesicle (v-SNAREs) and the target membrane (t-SNARES) are crucial to intracellular membrane fusion and protein and lipid traffic in Eukaryotes [8]. Since the hydrophobic heptad register of their α-helical coiled-coil region (SNARE motif) is interrupted at the so-called "zero layer" by a conserved R or Q residue, they are often referred to as R- or Q-SNAREs, respectively [9]. SNAREs can significantly vary in sequence length (e.g. from <100 amino acids of some synaptobrevins to >1100 residues among tomosyns) because of their modular domain architecture. In addition to SNARE motifs they can show further regions, such a carboxy (C)-terminal transmembrane region, motifs allowing post-translational addition of lipid anchors [8] and a variable or conserved amino (N)-terminal domain. The N-terminal Longin domain (LD) of longins [10] was found to play multiple regulatory roles (reviewed in [11]). Longins are the only R-SNAREs conserved in all Eukaryotes, whereas brevins are limited to bilateria and hence are absent from whole taxa (e.g., plants) [12]. Longins group into three subfamilies, prototyped by Ykt6, Sec22b and VAMP7 [11]. In yeast, the LD of Ykt6p can regulate membrane fusion by inhibiting Ykt6p participation to the fusion bundle, by competitive intramolecular binding to the SNARE motif [13]. The LD of VAMP7 also regulates membrane fusion; furthermore, it is crucial to neurite outgrowth, as overexpression of a "deregulated" fragment missing the LD (Δ-longin) increases neurite outgrowth whereas reverse effect (outgrowth inhibition) is obtained when expressing the LD alone [14,15]. In addition to regulating membrane fusion, LDs serve as a dominant signal for subcellular targeting. For example, in animals LD targets VAMP7 to late endosomes by binding to the δ-subunit of the AP-3 complex [16]. In plants, several VAMP7 proteins are targeted to their different subcellular localizations by their LDs [17]. The LD targets Ykt6 to its subcellular localization, likely by masking other localization signals [18]. In Sec22b, export from the endoplasmic reticulum is mediated by binding to Sec23/24, a process depending on a conformational epitope created by intramolecular LD-SNARE motif binding; such binding also results in preventing promiscuous, unspecific binding by sequestering the N-terminal half of the SNARE motif [19]. Also VAMP7 adopts a closed conformation based on intramolecular LD-SNARE motif binding [20]. Hrb, a clathrin adaptor and ArfGAP, binds the LD of VAMP7 by its unstructured SNARE-like motif, outcompeting the SNARE motif of VAMP7 for the same groove and suggesting that Hrb-mediated endocytosis of VAMP7 occurs only when this longin is incorporated into a *cis*-SNARE complex [21,22]. VAMP7 has been found to also interact with the positive regulator of neurite growth Varp, a guanine nucleotide exchange factor (GEF) of the small GTPase Rab21 and a binding partner of Rab 32 and Rab 38 [23,24].

Like other highly regulated processes typical of eukaryotic cells, subcellular trafficking needs fine tuning of protein functions and structures. Modulation of domain architecture and creation or deletion of sequence motifs can in turn be achieved by alternative splicing (AS): this way, a relatively large proteome can be created starting from a limited number of genes [25]. Indeed, AS has been shown to occur in several trafficking-related genes, including SNARE genes, resulting in the production of isoforms with different subcellular localization and trafficking properties. Developmentally regulated AS of SNAP-23 and SNAP-25 modulates interactions with accessory factors and subcellular localization [26,27]; AS also regulates interaction properties in the large t-SNARE family of syntaxins, by varying their N-terminal and/or C-terminal regions [28]. In R-SNAREs, AS can finely tune vesicle subcellular targeting by varying the extreme C-terminal region in splice variants of VAMP1 [29,30], and tissue-specific AS of mammalian tomosyn is involved in the regulation of neuronal secretion [31,32]. In most Eukaryotes, the involvement of VAMP7 in multiple trafficking events at different subcellular compartments is mediated by gene amplification and variation [33]. In mammals, VAMP7 is encoded by a single gene and yet it regulates multiple subcellular pathways [33,34]: hence, AS is a likely strategy to achieve such complexity in regulation. Thus, we have started investigation on longins (the most conserved R-SNAREs) by a thorough analysis for the occurrence of AS at the human gene SYBL1.

## Results and discussion

### Exon skipping at the SYBL1 locus results in modulating isoform domain architecture

**Figure 1:**
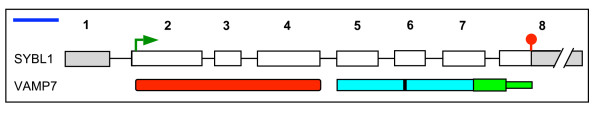
**Genomic structure of SYBL1 and domain architecture of VAMP7**. Top: exon-intron structure of the human gene SYBL1 (AJ004799). The 100 bp reference bar (blue, top left) only refers to exons; introns are not to scale. Exons are numbered: coding regions are white and non-coding ones are grey; start (green arrow) and stop (red circle) codons are indicated. Bottom: domain architecture of the encoded protein VAMP7 (P51809): Longin domain (red), SNARE motif (cyan; the black vertical bar indicates the conserved arginine of the polar layer) and transmembrane region (light green) with intravesicular tail (thin light green line).

Human gene SYBL1 is composed of eight exons, spanning a genomic region of approximately 35 kb [35]. Figure 1 shows that its genomic structure is intriguingly related to the domain architecture of VAMP7 (UniProtKB/Swiss-Prot accession P51809): the LD is encoded by exons 2 to 4, whereas exons 5 to 8 encode the helical domains (SNARE motif and transmembrane region). Within the literature two AS variants of VAMP7 have been described, the main isoform VAMP7a (P51809-1) and a variant, VAMP7c, missing part of exon 2 and exon 3 (P51809-3). We identified isoform VAMP7b, missing exon 6, during the screening of a cDNA library [1]. We deposited the RNA and protein sequences into database (accession numbers being respectively AJ295938 and P51809-2) and the characterization is reported in this work. Database searches allowed us to identify EST clone AW163735, likely corresponding to an uncharacterized splice variant missing exon 3, hereafter called VAMP7d. We then performed RT-PCR to unravel AS at the SYBL1 locus. Preliminary experiments with primers pairing to the untranslated regions of the gene amplified multiple fragments (not shown), the longest of which corresponding to VAMP7a; we found no intron retention, suggesting AS of SYBL1 is based on exon skipping. This prompted us to set up more specific and resolutive experiments, investigating 5' and 3' SYBL1 splicing events separately.

### Exon skipping at the 5' half of the gene results in synaptobrevin-like, "non-longin" isoforms

**Figure 2:**
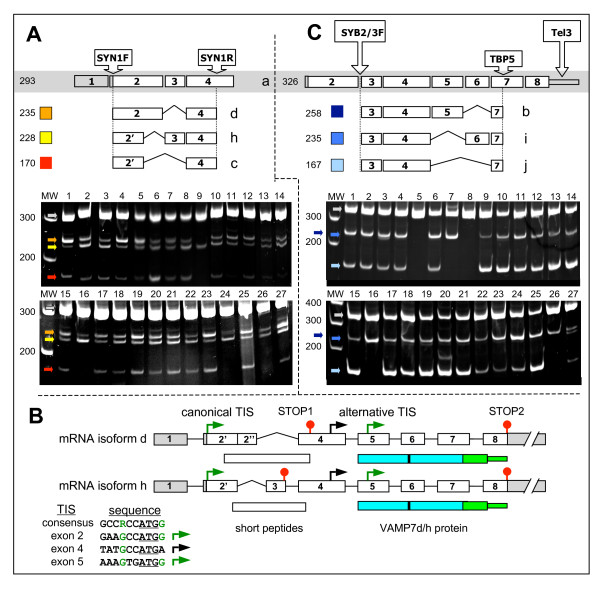
**RT-PCR analysis of alternative splicing of SYBL1**. Sizes (in base pairs) of MW markers and expected lengths of amplified fragments are reported. Isoforms and corresponding amplified fragments are color coded. Exonic structure of VAMP7a has grey background. The photographs show RT-PCR amplifications using cDNAs from tissues (1-19) or cell lines (20-27): 1, fetal brain; 2, total brain; 3, hippocampus; 4, cerebellum; 5, fetal heart; 6, heart; 7, skeletal muscle; 8, fetal liver; 9, liver; 10, mammary gland; 11, testis; 12, ovary: 13, pancreas; 14, kidney; 15, spleen; 16, colon; 17, small intestine; 18, stomach; 19, placenta; 20, T lymphocytes; 21, Raji; 22, HeLa; 23, RPE; 24, U937; 25, HEK293; 26, ND2; 27, ND2 15 days after induction. Panel A: variants missing the LD (VAMP7c, red; VAMP7d, orange; VAMP7h, yellow) were identified using SYN1F/R primers, placed on exon 2-containing ATG and exon 4. Panel B: structures of the VAMP7d and VAMP7h mRNAs and analysis of translation initiation sites (TIS). The nucleotide context for each TIS is aligned to the mammalian consensus (R, purine = A/G); nucleotides crucial to optimal initiation are green. Arrows indicate optimal (green) and non-optimal (black) TIS; red circles are stop codons. SNARE motif (cyan), conserved arginine (black vertical bar) and transmembrane region with intravesicular tail (light green) are also reported. Panel C: non-SNARE variants (VAMP7b, dark blue; VAMP7i, blue; VAMP7j, light blue) were identified by semi-nested RT-PCR with a VAMP7a specific ASOa primer (SYB2/3F) combined to a 3'UTR primer (Tel3) then to a nested primer (TBP5).

We first examined AS at the 5' end of the SYBL1 locus and identified three isoforms, "c", "d" and "h", that would disrupt production of the Longin domain (Figure 2A). AS at LD-codifying exons was firstly screened with the forward primer including the canonical ATG codon of the SYBL1 gene. Three bands smaller than isoform "a" were sequenced, of which one confirmed the bioinformatically captured isoform "d" missing exon 3. The two other bands resulted from the use of a cryptic GT donor splice site inside exon 2 joined to the AG acceptor splice site of either exon 3 (isoform "h") or exon 4 (isoform "c"). In order to characterize the 3' architecture of each isoform, we used an "allele specific oligonucleotide" PCR (ASO-PCR) approach [36]. ASO-c, ASO-d and ASO-h forward primers anneal specifically only to c- or d- or h-like isoforms (showing incomplete LD sequences), completely abolishing any other splice-molecule influence. Each of these ASO primers was combined to a common reverse primer specific to SYBL1-exon 8, performing four different sets of RT-PCR with cDNAs from different tissues and cell lines (Figure 2). We could conclude that "non-longin" isoforms - i.e. splice variants missing the LD - retain all the canonical exons from 4 to 8 (not shown), but with different consequences on the reading frame. VAMP7c was already described: the splicing event skips out 40 residues of the N-terminal region (which thus cannot fold as a LD), but the mRNA retains the original reading frame, leading to the correct translation of the "synaptobrevin-like" part of the sequence (including SNARE motif, transmembrane region and intravesicular tail) [16]. Translation of both VAMP7d and VAMP7h mRNAs would result in short peptides (59 and 45 aa, respectively) when starting from the canonical translation initiation start (TIS) of SYBL1, which is located in exon 2. However, scanning of the SYBL1 sequence (using StartScan [37]) identified a second, alternative TIS in exon 5, showing "optimal status" with respect to sequence context and distance from the upstream stop codons to reinitiate translation (Figure 2B). Such translation reinitiation mechanism is well-known in *Homo sapiens *and it allows for producing alternative, functional proteins from mRNAs which would otherwise originate only short peptides when using only the canonical TIS [38-42]. Since the alternative TIS in exon 5 is common to "d" and "h" isoforms, both mRNAs are likely to produce by translation reinitiation the same polypeptide, hence named "VAMP7d/h". Intriguingly, VAMP7d/h, corresponding to last 94 residues of VAMP7a, lacks the LD and consists only of the SNARE motif, transmembrane region and intravesicular tail, possibly representing a physiological equivalent of the "Δ-longin" recombinant VAMP7 construct enhancing membrane fusion and neurite outgrowth when expressed in neurons [15].

### Skipping of SYBL1 exons 5 and/or 6 produces non-SNARE, LD protein isoforms

**Figure 3:**
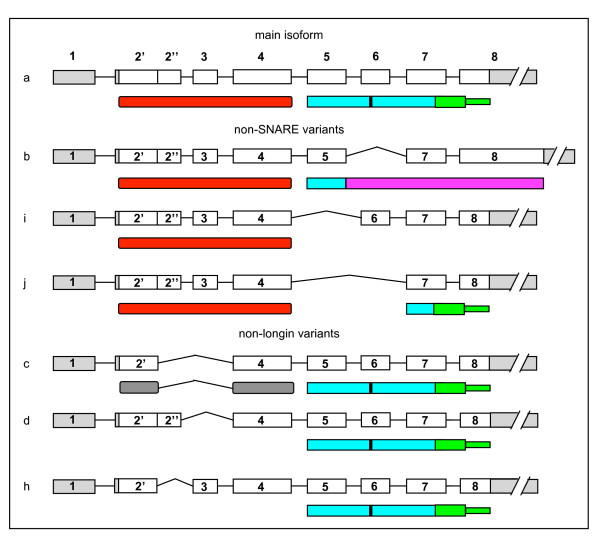
**Alternative mRNAs of the human SYBL1 gene and corresponding VAMP7 isoforms**. Coding exons or their fragments are white; non coding ones are grey. Introns are not to scale. Domain architecture: Longin domain, red; SNARE motif, cyan (the conserved arginine of the polar layer is indicated by a vertical bar); transmembrane region and intravesicular tail, light green; N-terminal region of VAMP7c, grey; unknown function region of VAMP7b, magenta.

Examining the 3' end of the gene we identified three splice variants, "b", "i" and "j" that result in proteins containing the LD but not the SNARE motif (Figure 2C). Such non-SNARE isoforms - characterized by skipping of respectively: (i) exon 6 (VAMP7b), (ii) exon 5 (VAMP7i) and (iii) both exons (VAMP7j) - show different domain architectures possibly underlying different cellular roles. VAMP7b is 40 amino acids longer than VAMP7a and it shares with the main isoform the LD and N-terminal part of the SNARE motif. Then, a frameshift downstream of exon 5 results in substitution of both the C-terminal half of the SNARE motif and the transmembrane region by a novel C-terminal region of 116 amino acids. On the other hand, VAMP7i basically consists of the LD alone: exons 1 to 4 encode for the first 114 residues of VAMP7, including all secondary structure elements needed to achieve a complete LD fold (according to PDB structure 2dmw). Skipping of exon 5 then results in a frameshift that leads to the translation of just two additional residues, followed by a premature stop codon. As for VAMP7j, it is a kind of "membrane-anchored version" of VAMP7i: skipping of both exons 5 and 6 preserves the original frame in which the LD is followed by a short hinge region preceding the original transmembrane and intravesicular tail regions, shared with the main isoform. Multiple skipping of exons 3 to 6 was found to result in a number of putative additional isoforms; however, multi-exon skipping often derives from splicing errors instead of programmed AS and "false" isoforms can be recognized by quite low expression levels [43]. Therefore, we performed semi-quantitative and quantitative RT-PCR screens and expression levels of these putative isoforms were found to be less than 0.1% (not shown) of the main isoform, as reported for splicing errors [43]. Even though the existence of further isoforms cannot be excluded, assays performed so far show that AS at the SYBL1 locus results in the production of seven isoforms with varying domain architecture (Figure 3).

### Quantitative expression and tissue specific distribution of VAMP7/TI-VAMP isoforms

**Figure 4:**
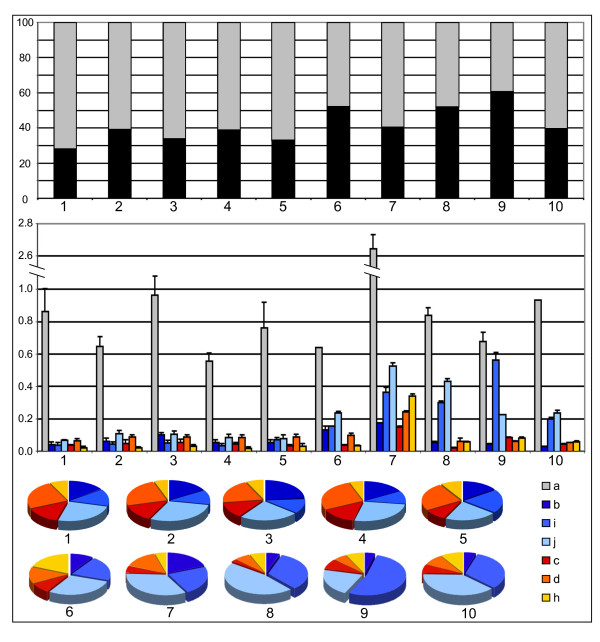
**Quantitative analysis of the VAMP7 isoform mRNA levels in human cell lines and tissues**. Color coding for all isoforms is reported; in the upper graph, 100% is the VAMP7 mRNA pool, of which the black part represents the pool of alternative isoforms (b+c+d+h+i+j) mRNA. In the middle, original data (mRNA levels of each isoform) are reported as % of the housekeeping reference gene (ribosomal S13) mRNA. Lower graphs compare individual levels within the pool of alternative isoforms (excluding VAMP7a). Cell lines (1-5) and tissues (6-10) are the followings: 1, C63; 2, HepG2; 3, HeLa; 4, Jurkat; 5, SH-SY5Y; 6, fetal brain; 7, adult brain; 8, colon; 9, skeletal muscle; 10, spleen.

Real-time RT-PCR was performed on a panel of human cell lines and tissues, in order to obtain amplicons specific to VAMP7 isoforms (see methods). Figure 4 summarizes quantitative expression data. The upper graph illustrates relative contributions to the mRNA pool from VAMP7a (grey) and alternative splice variants (black). VAMP7a contribution to the mRNA pool amounts to 60-70% in cell lines (samples 1-5) and 40-60% in tissues (samples 6-10); in three out of five tissues, the sum of alternative variants (b, c, d, h, i, j) overpass 50% of the VAMP7 mRNA. The central graph illustrates the mRNA levels of single isoforms; splice variants range 5-12% of VAMP7a in cell lines, while their levels are much higher in tissues. For instance, in colon (sample 8) the aggregate level of VAMP7i+j mRNAs is comparable to VAMP7a and in skeletal muscle (sample 9) VAMP7i alone is comparable to the main isoform. In pie charts, 100% refers to the mRNA pool of alternative variants, excluding VAMP7a. Preponderance of LD^+^, non-SNARE isoforms ("b", "i" and "j") is apparent in cell lines and it becomes more evident in tissues, where their aggregate level is comparable or even higher than that of VAMP7a. In particular, the non-SNARE/SNARE isoform ratio can shift from approximately 1:1 in cell lines to up 3:1 in tissues. In most tissues and cell lines, the second most expressed isoform (after VAMP7a) is VAMP7j, which is highly expressed in colon. The level of alternative variants in neuroblastoma cells (sample 5) and fetal brain (sample 6) is lower than that found in adult brain (sample 7), where the expression of VAMP7a is high (in agreement with literature [1]).

### Immunodetection of isoforms by domain-specific oligoclonal antibodies

**Figure 5:**
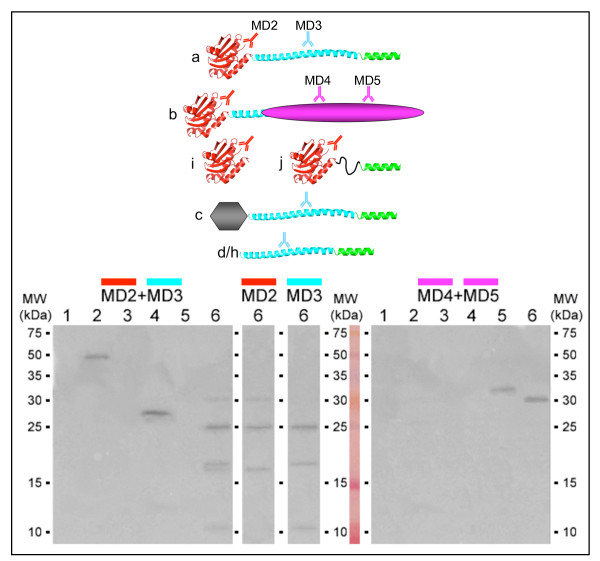
**Immunoblotting with domain-specific anti-peptide antibodies**. Each MD antibody shares the color with the recognized domains: MD2, Longin domain, red; MD3, SNARE motif, cyan; MD4 and MD5, unknown function region of VAMP7b, magenta. Other represented regions are the N-terminal region of VAMP7c (grey) and the transmembrane region with intravesicular tail (light green). The two large blots share the same series of whole lysates from *E. coli *cells (lanes 1-5) or Jurkat cells (lane 6) and each blot was incubated with antibodies indicated on the top. Bacterial cells express empty control plasmids (lane 1, pGEX; lane 3, pET32H), or the following VAMP7 fragments: cytoplasmic of VAMP7a fused C-terminally to GST in pGEX (lane 2), LD of VAMP7a fused C-terminally to thioredoxin in pET32H (lane 4), C-ter region of VAMP7b fused C-terminally to thioredoxin in pET32H (lane 5). Molecular weight markers are reported.

In order to get evidence on isoforms at protein level, oligoclonal antibodies were made to regions of VAMP7: MD2 recognizes just the LD, MD3 the C-terminal half of the SNARE motif, MD4 and MD5 the alternative non-SNARE C-terminal region of VAMP7b (see Figure 5 for domain and isoform recognition specificity). Specificity of each antibody was checked (see methods) and confirmed by complete absence of non specific recognition, even at high concentration (1:100); MD4 and MD5 yielded a stronger signal than MD2 and MD3. For immunoblotting experiments, two identical series of protein samples were separated by SDS-PAGE, blotted and incubated with antibodies specific to the LD and SNARE motif of VAMP7 (MD2 and MD3, which were used in combination, as oligoclonal antibodies are weaker than polyclonal ones) or to the unique C-terminal region of VAMP7b (MD4 and MD5). In extracts from bacteria expressing VAMP7 fragments, MD2+MD3 recognized bands of the expected M_R _of GST-cytoplasmic domain of VAMP7a (sample 2) and of thioredoxin-LD (sample 4); MD4+MD5 recognized only the band corresponding to thioredoxin-C-terminal region of VAMP7b (sample 5). Specificity was confirmed by the fact that no band could be seen in control extracts from bacteria expressing the empty plasmids (samples 1 and 3). Specificity of MD4+MD5 antibodies was expected to be high also when using human cell extracts, because exhaustive scanning of the human genome failed to find sequences with significant similarity to the C-terminal region of VAMP7b. Such prediction was confirmed by the fact that among endogenous human proteins (lane 6, right blot), MD4+MD5 recognized a single band of approximately 30 kDa (expected M_R _of VAMP7b). As expected, MD2+MD3 identified a main band of approximately 25 kDa (M_R _of VAMP7a) and the 30 kDa band corresponding to VAMP7b (lane 6, left blot). Recognition of VAMP7b by both antibody panels is in agreement with MD2 specificity to the LD, shared by isoforms "a" and "b". Intensities of the two 30 kDa bands are also in agreement with relative strengths of the antibodies (MD4+MD5 >> MD2+MD3). Moreover, relative intensities of the 25 kDa and 30 kDa bands in lane 6 (left blot) are in agreement with higher expression of the main isoform (as reported in Figure 4). Given that MD2+MD3 are expected to weakly recognize all splice variants reported here, additional bands in lane 6 (left blot) are likely to correspond to other isoforms. In particular, the two bands in between 25 kDa and 15 kDa markers are compatible with expected M_R _of VAMP7c (~20 kDa) and VAMP7j (~19 kDa) and the band slightly over the 10 kDa marker might correspond to VAMP7d/h (~10.5 kDa). To better identify the minor bands, the same sample (extract from Jurkat cell) was investigated using MD2 and MD3 antibodies separately (lanes 6 in between the two large blots). As expected, MD2 (specific to the LD) recognized - in addition to VAMP7a (25 kDa) and VAMP7b (30 kDa) bands - the 19 kDa band likely corresponding to VAMP7j. MD2 did not recognize the bands of ~20 and 10-11 kDa, suggesting that corresponding proteins lacked the LD epitope. This was confirmed by the somewhat "reverse" pattern seen when using the SNARE-specific antibody alone: in addition to VAMP7a, MD3 recognized the bands of ~20 kDa (expected M_R _of VAMP7c) and 10-11 kDa (expected M_R _of VAMP7d/h), while it did not recognize those putatively corresponding to isoforms VAMP7b and VAMP7j, both of which are missing the SNARE epitope. Absence of a band for VAMP7i is not surprising, given that this isoform shows low expression in Jurkat cells and it would be recognized only by the weakest antibody (MD2). Quantitative PCR data showed that in human tissues the expression of minor isoforms is relatively high. However, the MD antibodies could not be used with tissue extracts from other mammals, because AS at the SYBL1 locus shows organism-specific regulation, as e.g splice variants in human and mouse are different in both number and structure (M. Vacca, unpublished results). Therefore, further investigations will be needed to confirm the presence of all isoforms at protein level.

### VAMP7/TI-VAMP isoforms show different subcellular localizations

**Figure 6:**
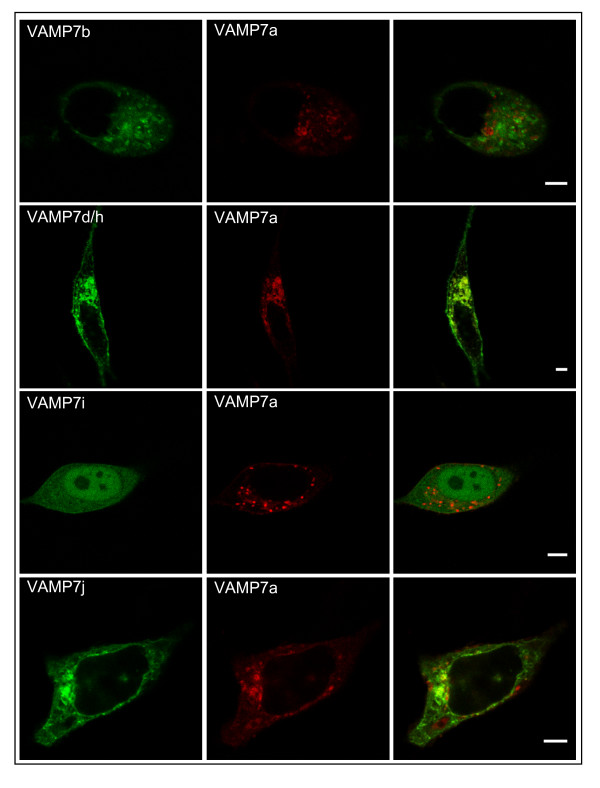
**Variation of subcellular targeting among VAMP7 isoforms**. Living HeLa cells, co-expressing an EGFP tagged splice variant of VAMP7 (left column) and the RFP tagged VAMP7a (central column). Merged images are presented on the right column. Reference white bars correspond to 5 μm. Confocal planes are presented.

The only VAMP7 splice variant characterized so far is VAMP7c: its truncated N-terminal region is unable to bind δ-AP3 and this results in a subcellular localization that differs from that of the main isoform [16]. Since the newly identified isoforms (b, d/h, i, and j) also show variant domain architectures, we compared their subcellular localization to the main isoform using fluorescent protein tagged chimeras. Figure 6 shows confocal plane images from living HeLa cells that were co-transfected with EGFP-tagged splice variant and RFP tagged main isoform. Large yellow areas of co-localization are apparent in merged images for VAMP7d/h. On the other hand, VAMP7b shows a somewhat widespread distribution and the subcellular localization of VAMP7j is also different from the punctate localization of VAMP7a, as red areas are clearly apparent in merged images. However, partial co-localization with VAMP7a cannot be excluded. Intriguingly, VAMP7i is the only isoform that also shows a nuclear localization. However, highly widespread VAMP7i and punctate, restricted-area VAMP7a indicate a quite different subcellular localization also in the cytoplasm.

## Conclusions

Fine tuning of gene functions is an important strategy by which higher organisms can achieve their complexity without needing a proportional increase in gene number. Indeed, AS at a single locus can result in the production of a (small to large) number of splice isoforms with varying domain architecture and function(s). It is noteworthy that increased complexity in trafficking routes mediated by VAMP7 is achieved in plants by increased gene number, whereas a corresponding increase in animals is not apparent. Rather, as observed for other SNAREs and trafficking proteins, the SYBL1 locus undergoes AS and this in turn results in a number of VAMP7 isoforms with intriguing modulation in domain architecture. Further work will be needed to characterize biochemical, structural and cellular properties of each isoform. However, these "natural mutants" of VAMP7 can be now used to shed more light on the function(s) of the LD and other regions/domains. To this aim, artificial constructs have been used so far: the subcellular localization of several chimeric VAMP7 proteins was compared in plants [17], whereas separated VAMP7 domains were used to investigate both subcellular localization and effects on mammalian cell physiology. In particular, a synaptobrevin-like Δ-longin fragment and an LD-only constructs were found to elicit somewhat opposite effects [14,15]. Isoforms VAMP7d/h and VAMP7i show the same domain architectures of respectively Δ-longin and LD-only fragments and are likely to mediate equal/similar effects. Noteworthy, VAMP7d/h and Δ-longin fragment co-localize with VAMP7a, while VAMP7i and LD-only do not, thus showing congruent subcellular sorting in two different cell types (neuronal and epithelial). AS at the SYBL1 locus results in a more sophisticated modulation of the domain architecture: two non-SNARE isoforms consist of the LD alone (VAMP7i) or "membrane-anchored" LD (VAMP7j), and two Δ-longin isoforms (VAMP7d/h, this work and VAMP7c, [16]) share synaptobrevin-like architecture with different N-terminal extensions. Finally, VAMP7b is only partially "non-SNARE" because it still keeps the N-terminal part of the SNARE, which is crucial for intramolecular binding to the LD, closed conformation and targeting [19]. VAMP7b and VAMP7a show different subcellular localization, in agreement with evidence that other protein regions/domains can modulate subcellular targeting capacity of the LD [21,22]. This is further suggested when comparing VAMP7i and VAMP7j, which share the LD and lack the SNARE motif. VAMP7i is found in the nucleus, whereas the transmembrane anchor in VAMP7j is likely to prevent nuclear localization. Indeed, VAMP7i consists only of the LD fold, similar to longin-like proteins [11] like σ AP [44] and sedlin [45]. Intriguingly, sedlin has been recently reported to show nuclear localization and to interact with transcription factors (TF) and TF-binding proteins [46,47]. Production by AS of non-SNARE isoforms further strengthens the concept that the LD can play an important role in trafficking independently on the SNARE motif, as suggested by the existence of non SNARE longins [33]. Tissue-specific expression data suggest that AS at the SYBL1 locus is developmentally regulated along cell lineage/tissue differentiation. Alternative mRNAs encoding variants with different domain combinations are expressed at levels ranging 5-12% of VAMP7a (Figure 4) in cell lines. In differentiated tissues the aggregate level of non-SNARE, inhibitory variants is comparable or even higher than VAMP7a. Each isoform might contribute to finely tune VAMP7-mediated regulation of cell physiology: indeed, several regulatory mechanisms do not require for 1:1 interactions. The main isoform is distributed among multiple locations along its trafficking route, because of interactions mediated by all its domains. Instead, splice variants missing either the LD or the SNARE motif are likely more involved in specific interactions, thus increasing their relative weight in influencing specific trafficking events. Evidence from this work further suggests that AS may contribute to finely tune the vesicle-mediated membrane trafficking specificity, providing new insights about the biological importance of the Longin domain over the SNARE functions.

## Methods

### Cell culture

The following human cell lines were used in this work: SH-SY5Y (neuroblastoma); C63 (skin fibroblasts); HepG2 (hepatocellular carcinoma); HeLa (cervical carcinoma); Jurkat (T-cell leukaemia). All cells were grown at 37°C in a humidified atmosphere, 5% CO_2_. SH-SY5Y and Jurkat cells were grown in the following media (all reagents from Invitrogen), supplemented with Glutamax and 10% heat-inactivated fetal bovine serum: SH-SY5Y, 1:1 DMEM+Ham's F12 supplemented with gentamicin (50 μg/ml); Jurkat, RPMI supplemented with 10 mM HEPES and PEN-STREP. C63, HepG2 and HeLa cells were kindly provided by colleagues from other laboratories.

### RNA extraction and retrotranscription

Total RNA from cell cultures was extracted using TRIzol (Invitrogen) according to manufacturer's instructions. Aliquots (5 μg) of DNase I-treated total RNA were reverse transcribed using SuperScript^® ^III RNase H-reverse transcriptase (Invitrogen). Total RNA from human tissues was purchased from BD Clontech. Aliquots (1 μg) of total RNA from human tissues were reverse transcribed using QuantiTect^® ^reverse transcription kit (Qiagen).

### Non quantitative PCR

PCR experiments were performed using Ampli Taq Gold DNA Polymerase (Applied Biosystems), with buffer recommended by the manufacturer, in a final reaction volume of 10 μl. An Express (Thermo-Hybaid) thermal cycler was used for the analysis of the 5' half of SYBL1 gene, set as follows: 9' at 96°C followed by 35 cycles consisting of 30" melting at 95°C + 45" annealing at 58°C + 45" extension at 72°C; a final extension step at 72°C was then performed for 5'. The forward (F) and reverse (R) (MWG)-primer sequences were: SYN1F 5'-ATGGCGATTCTTTTTGCTGTTGTT-3'; SYN1R 5'-AGTGCTGTCTGTGCTCTTGAACCGT-3'. The 3' architecture of LD+ isoforms was investigated carrying out a seminested PCR in the Robocycler (Stratagene) with an annealing temperature of 55°C and steps of annealing and extension of 1'. In both PCRs the same forward primer, SYBL2/3F, was used: 5'-CGTACTCACATGGCAATTAT-3'. This forward primer (ASOa) is specific to VAMP7a; it was combined to the TEL3 reverse primer: 5'-AGTCACATGGATTGCTTTTA-3' in the first PCR round (28 cycles) and to the TBP5: 5'-CAAGATTTCTGCTGGTAG-3' in the second PCR round (35 cycles) as nested reverse primer.

### Real-time RT-PCR

All experiments were performed as follows: RNA for each sample was extracted and reverse transcribed in three replicates. In order to minimize variation depending on experimental error, real-time experiments were then performed in five replicates for each sample, resulting in 3x5 = 15 replicates. Real-time PCR experiments were performed using SYBR^®^-Green core reagent kit and Ampli Taq Gold DNA Polymerase (Applied Biosystem) in a final reaction volume of 25 μl. The thermal cycler (Rotor-Gene 3000 from Corbett Research) was set as follows: 9' at 95°C followed by 40 cycles consisting of 30" melting at 95°C + 30" annealing at 60°C + 35" extension at 72°C. The gene encoding human ribosomal protein S13 is the housekeeping control for normalization [48]. For quantitative data analysis, the Rotor Gene software (version 6.0.34) was used according to Pflaffl [49] and Marino *et al. *[50]. The following F and R primers (SIGMA-Genosys) were used:

VAMP7a-F: 5'-CACTGATGATGATTTTGAACG-3';

VAMP7a-R: 5'-CTGAGCTACCAGATCTATGTTTCT-3';

VAMP7b-R: 5'-TTGAAGGTGACAGACTATGTTTC-3';

VAMP7c-F: 5'-CTTCCTGGAGGATTTTGAAC-3';

VAMP7d-F: 5'-CACATGGCAAGATTTTGAAC-3';

VAMP7h-F: 5'-GGAAACTTCCTGGAGTTATTTG-3';

VAMP7h-R: 5'-TCTCCATCACTTTGTCTAGGC-3';

VAMP7i-F: 5'-GCAGATTCTGGCTAAGATACC-3';

VAMP7i-R: 5'-TACCAGATCAGCTGTGCAG-3';

VAMP7j-F: 5'-ACAGCTGTCTGTCACCTTC-3';

VAMP7j-R: 5'-TCAGTATGTGAAAGGCTTGAG-3';

S13-F: 5'-TACAAACTGGCCAAGAAGGG-3';

S13-R: 5'-GGTGAATCCGGCTCTCTATTAG-3'.

Primers were designed and combined so that all amplicons had (or almost) equal length (250 to 254 bps) hence quantitative comparison could be extended to different amplicons from all isoforms. Primer combinations to amplify specific isoforms were: VAMP7a, VAMP7a-F + VAMP7a-R; VAMP7b, VAMP7a-F + VAMP7b-R; VAMP7c, VAMP7c-F + VAMP7a-R; VAMP7d, VAMP7d-F + VAMP7a-R; VAMP7h, VAMP7h-F + VAMP7h-R; VAMP7i, VAMP7i-F + VAMP7i-R; VAMP7j, VAMP7j-F + VAMP7j-R.

### Recombinant proteins

Plasmid pGEX expressing the cytoplasmic domain of VAMP7a fused to the N-terminal GST tag was a kind gift of Dr. Monica Cuccurese; the LD of VAMP7a/b (aa 1-118) and the novel C-terminal region of VAMP7b (aa 151-260) were cloned in a pET32(a) plasmid (Novagen) and expressed in *E. coli *BL21 cells following standard protocols.

### Oligoclonal antibodies and immunoblotting

Whenever possible, peptide sequences were designed to start N-terminally by a Cys residue (for conjugation to the carrier protein) already present in the VAMP7 isoform sequences:

MD2 (CITDDDFERSRAFNFLNE; residues 64-81 of VAMP7a and VAMP7b)

MD3 (Cys-RGERLELLIDKTENLVD; residues 150-166 of VAMP7a)

MD4 (CSSHVYEEPQAHYYH; residues 156-170 of VAMP7b)

MD5 (CDSSLSHTDRWYLPV; residues 219-233 of VAMP7b)

Peptide synthesis and conjugation, immunisation of rabbits and sera collection were performed by a company (Sigma-Genosys); we then purified the antibodies by affinity chromatography. Peptides were Cys-conjugated to the Sulfolink resin and chromatography was performed using binding and elution buffers from Pierce, following manufacturer's instructions. After elution, antibodies were concentrated by ultrafiltration using 10 kDa cut off filter tubes (Amicon). Strength and specificity of each antibody was checked by dot-blot analysis, using as targets progressive dilutions of (i) each specific peptide, (ii) the other three VAMP7 peptides and (iii) peptides from non-homologous proteins. For immunoblotting, protein samples were separated by standard SDS-PAGE and blotted onto nitrocellulose filters using Protean and Transblot cells (Bio-Rad). Primary antibodies were diluted 1:1000; secondary antibody (anti rabbit IgG-HRP, Santa Cruz Biotechnology) was diluted 1:20000. Enhanced chemiluminescence detection was performed using the Supersignal system (Pierce), following manufacturer's instructions; blot images were acquired using a Chemi-Doc image analysis system (Bio-Rad).

### Subcellular localization experiments

The main isoform and splice variants of VAMP7 were tagged with a fluorescent protein (either red, RFP, or green, EGFP) cloning the isoform cds in pRFP-C3, pEGFP-C3 or pEGFP-N1 (Invitrogen). VAMP7b has C-terminal tag, whereas VAMP7d/h, VAMP7i and VAMP7j have N-terminal tags.

HeLa cells were transiently cotransfected using Lipofectamine™2000 protocol (Invitrogen) with pRFP-C3-VAMP7a and pEGFP-N1-VAMP7b or pEGFP-C3-VAMP7dh, pEGFP-C3-VAMP7i, pEGFP-C3-VAMP7j. Then, *in vivo *analysis was performed at 15/22/24/48h by using a TCS SP2 confocal scanning microscope (Leica, Heidelberg, Germany), sequential excitation with 488 nm, 543 nm laser beams, 63X 1.4 NA lens (low magnification: zoom 2-3; high magnification: zoom 5-8) and LAS AF Software. Images (size set to 512x512 pixels) were assembled by using ImageJ.

### Bioinformatics

Melting temperatures of PCR primers were calculated using DNA Calculator http://www.sigma-genosys.com/calc/DNACalc.asp; possible occurrence of hairpins, self- and hetero-dimers was ruled out by Oligo analyzer 3.1 http://eu.idtdna.com/analyzer/applications/oligoanalyzer/default.aspx and the specificity was checked with MFEprimer http://biocompute.bmi.ac.cn/MFEprimer/[51], scanning both genomic and transcriptomic human databases. Translation Initiation Site (TIS) prediction was performed using StartScan http://bioinformatics.psb.ugent.be/webtools/startscan/[42]; *in silico *translation of coding sequences resulting from likely alternative start codons was obtained using the Traslate tool at the ExPAsy server http://www.expasy.ch/tools/dna.html.

Antigenic regions of VAMP7 isoforms were predicted based on the antigenic index plot obtained using the Protean module of the Lasergene Package (DNASTAR); selection of peptide sequences was based on both their conservation/specifity and predicted solubility.

## Authors' contributions

MV identified most of splicing isoforms, LA performed expression and confocal microscopy analyses, FDR designed and took care of cloning steps, AC designed and coordinated expression analyses, VR purified the antibodies, performed immunological and expression analyses, MS identified VAMP7b, NDF performed TIS analysis and participated to expression studies, OR coordinated cell biology tasks, FF and MDE coordinated the whole work and wrote the manuscript. All authors read and approved the final manuscript.
